# Disentangling complex parasite interactions: Protection against cerebral malaria by one helminth species is jeopardized by co-infection with another

**DOI:** 10.1371/journal.pntd.0006483

**Published:** 2018-05-10

**Authors:** Jessica L. Abbate, Vanessa O. Ezenwa, Jean-François Guégan, Marc Choisy, Mathieu Nacher, Benjamin Roche

**Affiliations:** 1 UMMISCO, IRD / Sorbonne Université, Bondy, France; 2 MIVEGEC, IRD, CNRS, Université Montpellier, Montpellier, France; 3 Odum School of Ecology and Department of Infectious Diseases, College of Veterinary Medicine, University of Georgia, Athens, GA, United States of America; 4 Oxford University Clinical Research Unit, Hanoi, Vietnam; 5 CIC INSERM 1424, Centre Hospitalier de Cayenne, Cayenne, French Guiana; 6 EA3593, Ecosystèmes Amazoniens et Pathologie Tropicale, Université de Guyane, Cayenne, French Guiana; 7 Departamento de Etología, Fauna Silvestre y Animales de Laboratorio, Facultad de Medicina Veterinaria y Zootecnia, Universidad Nacional Autónoma de México (UNAM), Ciudad de México, México; Emory University, UNITED STATES

## Abstract

Multi-species interactions can often have non-intuitive consequences. However, the study of parasite interactions has rarely gone beyond the effects of pairwise combinations of species, and the outcomes of multi-parasite interactions are poorly understood. We investigated the effects of co-infection by four gastrointestinal helminth species on the development of cerebral malaria among *Plasmodium falciparum*-infected patients. We characterized associations among the helminth parasite infra-community, and then tested for independent (direct) and co-infection dependent (indirect) effects of helminths on cerebral malaria risk. We found that infection by *Ascaris lumbricoides* and *Trichuris trichiura* were both associated with direct reductions in cerebral malaria risk. However, the benefit of *T*. *trichiura* infection was halved in the presence of hookworm, revealing a strong indirect effect. Our study suggests that the outcome of interactions between two parasite species can be significantly modified by a third, emphasizing the critical role that parasite community interactions play in shaping infection outcomes.

## Author summary

Over the last decade, an increasing number of studies have shown that co-infected individuals can show strikingly different disease symptoms than singly infected individuals. Among these studies, the potential protective effect of some helminth species on malaria severity has attracted considerable attention because of the possible public health impact. However, despite the fact that helminth species frequently co-occur together within an individual, no studies have investigated the role of within-host helminth community interactions on malaria severity. Taking the advantage of a cohort study in Thailand, we found that infection by *Ascaris lumbricoides* and *Trichuris trichiura* were both associated with direct reductions in cerebral malaria risk. However, the benefit of *T*. *trichiura* infection was halved in the presence of hookworm, revealing that interactions among helminth species can jeopardize the beneficial role of individual helminths. Our study shows that the outcome of interactions between two parasite species can be significantly modified by a third, emphasizing the critical role that parasite community interactions play in shaping infection outcomes and their impact on public health. These insights are especially relevant for neglected tropical diseases that occur in areas of high pathogen diversity.

## Introduction

Most hosts are co-infected by a range of pathogenic organisms (e.g., [1]), and interactions between co-infecting parasites can be important determinants of susceptibility to infection (e.g., [2]) as well as the severity of disease symptoms (e.g., [3]). Co-occurring parasites interact within a host via a number of mechanisms, including through active down-regulation of immune responses by some parasites, indirect competition through stimulation of host defenses, and direct competition for resources [4]. Importantly, the resulting impacts of within-host parasite interactions on host health can be beneficial (e.g., cross-protection between influenza strains, [5]) or detrimental (e.g., stimulation of the immune system’s response against many helminth species reduces the capacity of vertebrate hosts to respond to some intracellular pathogens [6]).

The majority of ecological studies on co-infection have focused on understanding the ecological, epidemiological, and evolutionary consequences of pairwise parasite interactions (e.g., [7–12]). However, since the mechanisms by which any two parasites can interact are not mutually exclusive, the costs of co-infection to host health may frequently be offset by simultaneous benefits or influenced by other co-infecting parasites [13]. In community ecology, a rich body of literature on direct and indirect effects of multi-species interactions indicates that interactions between multiple species in a community can be additive, non-additive, or lead to entirely non-intuitive “ecological surprises” (e.g., [14–18]). Yet, remarkably few studies have looked beyond direct pairwise parasite-parasite interaction effects on disease (but see e.g., [19–21]).

Interactions between helminths and human malaria (particularly that caused by *Plasmodium falciparum*) are among the most well-studied pairwise parasite interactions [22]. Both experimental studies in rodents and cross-sectional studies in humans suggest that immunosuppressive effects of some helminth species can weaken host defenses against *Plasmodium spp*., resulting in increased malaria incidence and severity, while co-infection with other helminth species is associated with protective effects against cerebral malaria, the most lethal form of malaria (reviewed in [23,24]). However, in addition to interactions between helminths and *Plasmodium*, different helminth species can also affect one another [25,26]. For example the trematode *Schistosoma mansoni* has been shown to impair normal clearance of *Echinostoma revolutum* (another trematode) in experimentally co-infected lab mice [27]. In humans, there is also correlative evidence that *A*. *lumbricoides* and hookworm, two soil-transmitted intestinal helminths known to interact with *Plasmodium*, may themselves interact [28]. Thus, it is possible that the effects of any single helminth species on malaria might be significantly modified in the presence of other helminths in the system.

Given the possible web of complex interactions that can occur in the helminth-*Plasmodium* system, we tested the hypothesis that interactions between helminth species can modify the impact of single-species helminth infections on malaria severity. Taking advantage of a dataset on 283 *Plasmodium*-infected patients from western Thailand, who were screened for helminth infections and monitored for the development of cerebral malaria [29,30], we combined standard logistic regression analysis with an association screening approach (SCN; [31]), to characterize how both direct (independent) and indirect (co-infection dependent) effects of helminth species influence malaria severity. To do this, we first identified and quantified the types of co-infections occurring between four different helminth species in our study population, and then tested for significant interspecific associations. Next, we evaluated the impact of single helminths, and their interactions with one-another, on the development of cerebral malaria. By taking advantage of the well-described helminth-malaria co-infection system to investigate how helminths interact with malaria severity both singly and in combination, we provide new insight into the consequences of parasite interactions.

## Materials and methods

### Patient data

Passive surveillance data on malaria patients were collected from the Hospital for Tropical Diseases, Bangkok, Thailand, and are described in detail elsewhere [29,30]. The study was approved by the Ethical Committee of the Faculty of Tropical Medicine, Mahidol University and all data analyzed were anonymized [30]. A total of 283 individuals with hyperparasitemic malaria (high *P*. *falciparum* biomass, defined as parasitemia >5% or >200,000/μl, or presence of schizonts in a peripheral bloodsmear) were screened for the development of cerebral malaria (neurological dysfunction typically leading to death or disability) and the presence of four helminth species: *Ascaris lumbricoides* (roundworm, AL), *Trichuris trichuira* (whipworm, TT), hookworm (HW, primarily *Necator americanus*, but also including other species such as *Ancylostoma duodenale*), and *Strongyloides stercoralis* (threadworm, SS) in fecal samples. Experienced microscopists were responsible for screening for both *Plasmodium* (using thick and thin blood smears stained with 10% Giemsa and read at 1000x magnification) and gastrointestinal nematode species via egg morphology (also using standard light microscopy techniques). Sex and age class (<20 years old, 20–40 years old, and > 40 years old) were also recorded. Out of the 283 hyperparasitemic cases, 67 developed cerebral malaria, while the remaining 216 did not. The demographic composition of the study population is provided in Table 1.

**Table 1:** Demographic composition of study population. There were slightly more females in the <20 age group (35%) than in those 20–40 (23%) or over 40 (24%), but this was not statistically significant (*χ*^*2*^ = 4.3, df = 2, p = 0.12).

		Cerebral malaria	Hyperparasitemic controls
Sex			
	Female	14 (20.9%)	62 (28.8%)
	Male	55 (79.1%)	153 (71.2%)
Age			
	Median [range]	25 [14–72]	23 [3–74]
	< 20	15 (22.3%)	73 (33.8%)
	20–40	44 (65.7%)	123 (56.9%)
	> 40	8 (11.9%)	17 (7.9%)

### Overview of the analytical approach

Given that co-infection by multiple helminth species commonly occurs, univariate analysis of each species’ impacts on the development of cerebral malaria may be confounded by the patient’s co-infection status. Furthermore, facilitative or competitive interactions among helminth species would mean that occurrences of interacting parasites in any given patient are not independent of one-another. To address this issue, we first identified co-infection frequencies and quantified associations between helminth species using association screening (SCN) analysis, a method recently developed to identify complex association patterns in large parasite assemblages [31], combined with a rarefaction analysis. This approach allowed us to understand how single and multiple-species infections were distributed among patients and to infer the relative strength of associations identified as significant. We then tested for direct and indirect effects of each helminth species on the development of cerebral malaria using logistic regression analysis, correcting for co-infection status with analysis of deviance and explicitly including all possible interactions terms in the model. We used a model selection approach to identify the helminth interactions that had significant consequences on the development of cerebral malaria.

### Quantifying associations between helminth species

To identify potential interactions occurring between all possible combinations of the four helminth species in our dataset, we used SCN analysis [31] to quantify the absolute and relative strength of species associations in the dataset. The utility of this method is that in addition to identifying multi-species associations with a high degree of power, it identifies the precise nature of these associations (i.e. whether it is the presence or absence of each species that occurs more often than expected by chance). The absolute strength of an association was determined by the SCN analysis itself, applied as a p-value (described below). As strong associations are likely to have identical p-values, the relative strength of an association was determined by running the SCN analysis on systematically jackknifed subsets of the data (also described below), with the assumption that the detection of stronger associations will be less influenced by sampling biases. Given the prevalence of each parasite species in the study population, SCN analysis generates a simulation-based 95% confidence envelope around the expected frequency of each possible combination of concurrent infection status (a total of 2^NP^ combinations, where NP = the number of parasite species) under the null hypothesis of random parasite associations. Observed co-infection combinations falling above or below this envelope are considered to occur more or less frequently, respectively, than in 95% of the random simulations. Significance of the association is given as a *p*-value, calculated as the number of instances in which the simulated co-infection frequency differed (above or below the upper or lower threshold, respectively) from the observed frequency divided by the total number of simulations, and is thus dictated by the variance in the simulated frequencies. Power tests on this method revealed that there should be at least as many host individuals in the dataset as the number of possible parasite species combinations (2^NP^), and that stronger associations had lower *p*-values [31].

We tested the robustness of each association to sampling biases by rarefaction analysis, where the SCN analysis was performed on progressive sub-sets of the data: 10 partitions sampling from 10% to 100% of the data (see Supplementary Material S1 Appendix). For each partition, the SCN analysis was performed on 20 independent sub-samples; and associations were deemed ‘significant’ if the *p*-value was below 0.05 in at least 95% of the runs. A robustness score (RR) was given to each species combination, ranging from 0 to 10, representing how much data sub-setting an association could tolerate before it was no longer detectable. A score of RR = 1 indicates that 100% of the data are needed, RR = 10 indicates that the association was detected in all 10 partitions. Stronger associations will have higher RR scores; thus, RR scores provide a quantitative measure of the relative strength of significant associations which often have similar *p*-values.

### Examining direct and indirect effects of helminths on cerebral malaria

Direct effects of infection by each helminth species on the development of cerebral malaria, and indirect effects of interactions between helminth species, were tested using logistic regression with backwards stepwise removal of higher-order interaction terms. Analyses were implemented using the ‘glm’ function from the *stats* package in R [32] with a binomial logit link. The ‘step’ function was used for backward model selection to reduce the number of interactions in the final model by minimizing the relative Akaike information criterion (AIC) value. On this final model, *χ*^*2*^ analysis of deviance was performed to test each helminth species’ impact on cerebral malaria by first correcting for patient sex, age class, and co-infection status (using the ‘drop1’ function).

## Results

### Helminth infection and co-infection frequencies

Out of the 283 hyperparasitemic malaria patients screened, 139 (49.1%) were infected with at least one helminth species, including 20 (29.8%) of 67 that developed cerebral malaria. The most prevalent helminth infection was HW (infecting 30.1% of all patients), followed by TT (24.7%), AL (19.1%), and SS (10.6%). Half (49.6%) of all patients infected with helminths were infected with more than one helminth species, including 4 individuals who were co-infected with all four species (Table 2). The distribution of these co-infections among patients that did or did not develop cerebral malaria is given in S1 Table.

**Table 2:** Association screening (SCN) analysis results for all detected gastrointestinal helminth species infecting hyperparasitemic malaria patients. The observed (Obs) frequency of each co-infection status is given along with the lower (LL) and upper (UL) limits of the 95% confidence envelope. Robustness scores are given for significant associations (SCN *p*<0.05 for at least 95% of runs, see main text). SCN *P*-values reported are the maximum *p*-value returned by the SCN analysis when 100% of the data are sampled. Significant or trending associations are highlighted in bold for emphasis.

SCN and Rarefaction Results					All hyperparasitemic malaria patients (N = 283)												
Coinfection Status	Pathogen species present				Obs	LL	UL	Direction	Percentage of data sampled							Robustness Score	SCN p-value < =
									10% →100%								
4 species	**AL**	**TT**	**HW**	**SS**	**4**	**0**	**3**	**Frequent**	** **	** **	** **	** **		**✓**	**✓**	**2**	**0.0032**
3 species	**AL**	**TT**	**HW**		**17**	**0**	**10**	**Frequent**	**✓**	**✓**	**✓**	**✓**	**✓**	**✓**	**✓**	**7**	**1.0 x 10**^**−8**^
	AL	TT		SS	1	0	5	Rare								0	0.6410
	AL		HW	SS	2	0	5	Rare								0	0.7676
		TT	HW	SS	3	0	7	Random								0	0.5556
2 species	AL	TT			11	1	18	Frequent								0	0.4520
	AL		HW		9	3	21	Rare								0	0.7276
	AL			SS	1	0	9	Rare								0	0.4120
		TT	HW		14	5	28	Rare								0	0.9068
		TT		SS	1	0	11	Rare								0	0.1692
			HW	SS	6	0	14	Random								0	0.9600
Single infections	**AL**				**9**	**13**	**41**	**Rare**			**✓**	**✓**	**✓**	**✓**	**✓**	**5**	**0.0012**
		**TT**			**19**	**20**	**52**	**Rare**				**✓**	**✓**	**✓**	**✓**	**4**	**0.0036**
			**HW**		**30**	**30**	**64**	**Rare**					**✓**	**✓**	**✓**	**3**	**0.0132**
				SS	12	4	24	Rare								0	0.9960
Not infected	* *	* *		* *	**144**	**84**	**132**	**Frequent**	**✓**	**✓**	**✓**	**✓**	**✓**	**✓**	**✓**	**7**	**0.0004**

### Associations between helminth species

A number of significant associations between helminths were identified via SCN and rarefaction analysis. The most robust result was that three-way co-infections between AL, TT, and HW, occurred more often than expected by random chance (*p*<0.00001, RR = 7), indicating a strong positive 3-way association between these species (Table 2). Uninfected individuals were also highly over-represented in the dataset (*p* = 0.0004, RR = 7), while AL, TT, and HW were all found to occur alone significantly less often than expected by chance (Table 2). The weakest significant result suggested that all four parasites (Al, TT, HW, and SS) were involved in more frequent co-infection than expected by chance (*p* = 0.0032, RR = 2; Table 2). However, given the relative strength of the 3-way association between AL, TT, and HW and the random occurrence of solo-SS infections (*p* = 0.996, RR = 0), it is possible this result simply indicates that the mechanism responsible for the 3-way association between AL, TT, and HW operates whether or not SS is present. Moreover, the low observed frequency of 4-way co-infections (n = 4) accounts for the low robustness score, further indicating a lack of strong support for this result. Restricting the analysis to males, females, or the most co-infected age group (15–20 year-olds, S2 Appendix) resulted in qualitatively similar outcomes.

### Direct and indirect effects of helminths on cerebral malaria

**Fig 1:**
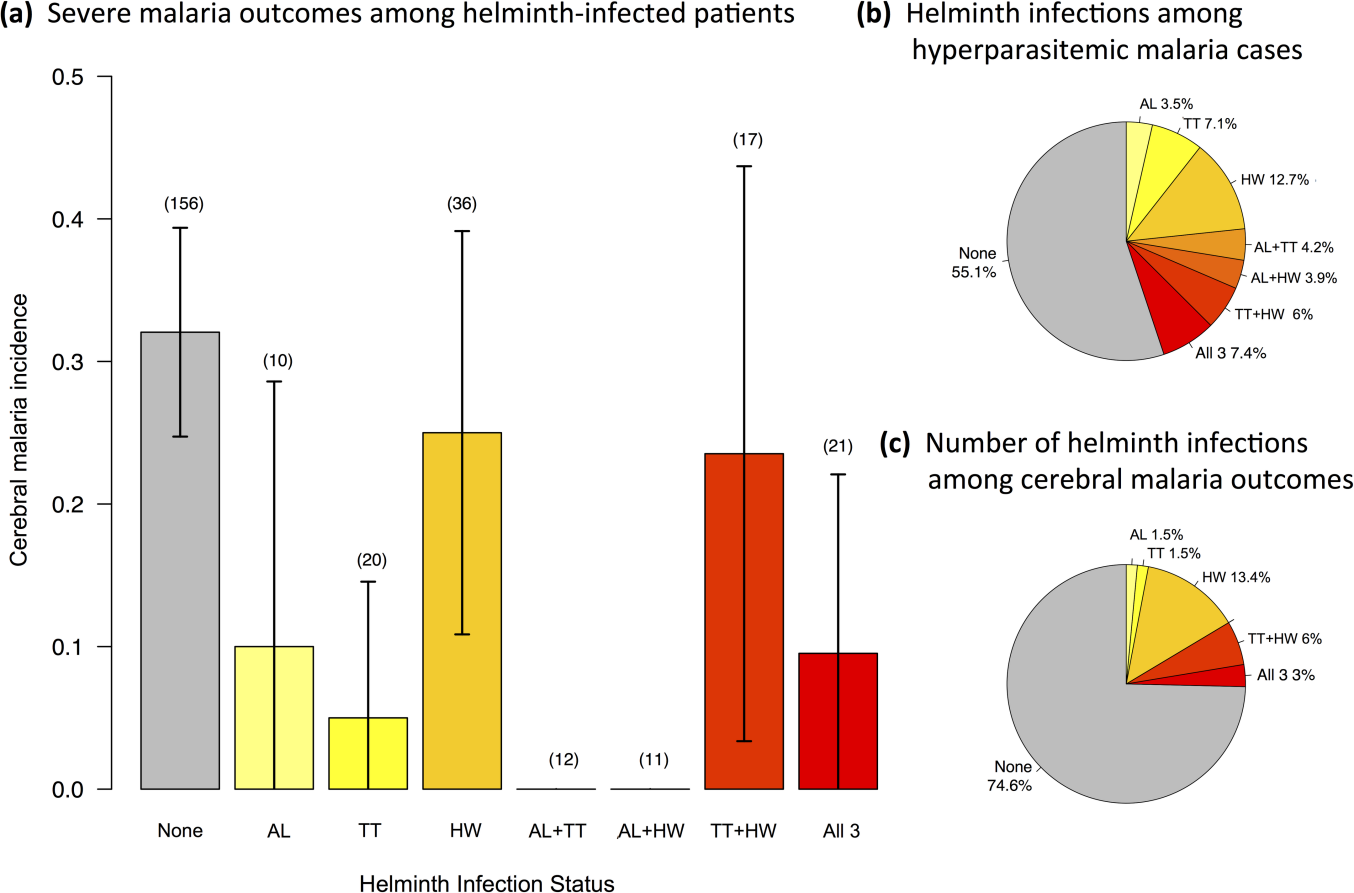
Malaria severity as (a) the proportion of cerebral outcomes for hyperparasitemic *P*. *falciparum* malaria cases (incidence) across patients with and without infection by helminths identified to be associated with clinical outcome: *A*. *lumbricoides* (AL), *T*. *trichiura* (TT), and hookworm (HW). Helminth infection statuses are mutually exclusive. The number of patients with each (co-)infection status is shown in parentheses above the error bars. Error bars represent 95% confidence interval of the proportion. Pie charts illustrate the relative contribution of each (co-)infection status to (b) the overall number of hyperparasitemic cases and (c) those that developed cerebral malaria.

**Fig 2:**
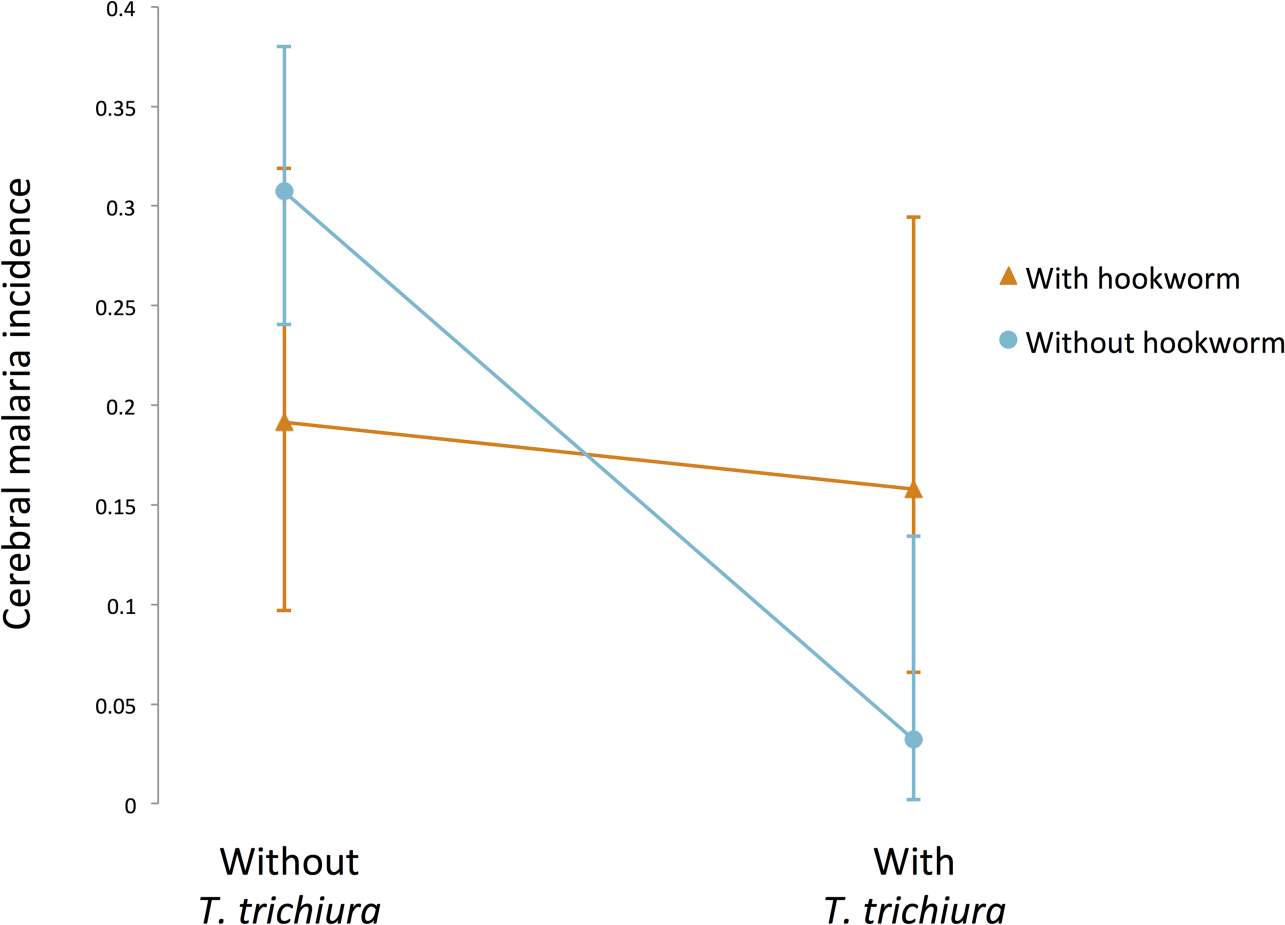
Significant interaction between *T*. *trichiura* and hookworm infection on development of cerebral malaria. Error bars represent 95% confidence intervals around the proportions. Cerebral malaria incidence (proportion of hyperparasiteic *P*. *falciparum* cases that developed cerebral malaria) is reported here without considering other co-factors or co-infections (*χ*^*2*^ = 5.56, p = 0.018).

We tested for both main (direct) and interaction (indirect) effects of helminths on cerebral malaria. We evaluated the main effects of all four helminth species, as well as all 2- and 3-way interactions for AL, TT, and HW. We excluded SS from interaction terms due to its low overall prevalence and low occurrence in helminth co-infections (with just 4 or fewer co-occurrences with any species other than hookworm, S1 Table), leading to insufficient statistical power. This decision was additionally supported by multiple correspondence analysis which indicated that variance in the development of cerebral malaria was associated with less than 8% of the total variance in SS occurrence (all single and co-infections combined) in our study group (S3 Appendix). Our full model testing the effect of helminths on cerebral malaria also included sex and age class as cofactors. Non-significant interaction terms were removed from the full model (AIC = 301) via backwards stepwise selection to achieve the final model shown in Table 3 (AIC = 292). Sex and age class, the direct effects of all four helminth species, and a single two-way interaction between TT and HW were all retained in the final model (Table 3). Both AL and TT were significant and independent predictors of cerebral malaria occurrence, and both were associated with protection from cerebral malaria (Table 3; Fig 1). Infection by AL reduced the odds of developing cerebral malaria by over 80%, while infection by TT was associated with a 90% reduction in cerebral malaria. HW and SS had no significant direct effects on cerebral malaria; however, HW had a significant indirect effect on the development of cerebral malaria via its interaction with TT. Co-infection by HW significantly reduced the protection offered by TT (OR: 12.39 [1.57 − 267.58], *p* = 0.015; Table 3). Patients infected with neither TT nor HW developed cerebral malaria at a rate of 30.7%, but this fell to 3.2% for patients infected with TT only. However, when patients were infected with both TT and HW the cerebral malaria infection rate rose to 15.8%, which reflects a 46% reduction in the protection offered by TT (Fig 2).

**Table 3:** Logistic regression (analysis of deviance and odds ratios) for analysis of helminth co-infection association with the risk of developing cerebral malaria. Statistical significance for each explanatory variable and interaction terms were determined by log-likelihood ratio tests after correcting for all other factors in the model. Interaction terms with *p*-values above 0.1 were removed in reverse step-wise procession.

		Adjusted odds of developing cerebral malaria			
	Estimate	95% CI	Deviance χ2	*p*-value	
Intercept	0.324	[0.14 − 0.75]			
Sex (F -> M)	0.921	[0.64 − 1.3]	0.21	0.64	
Age	1.013	[0.99 − 1.04]	0.93	0.33	
***A*. *lumbricoides* (AL)**	**0.211**	**[0.05 − 0.67]**	**7.34**	**0.007**	**
***T*. *trichiura* (TT)**	**0.106**	**[0.01 − 0.54]**	**8.63**	**0.003**	**
Hookworm (HW)	0.632	[0.26 − 1.39]	1.25	0.26	
*S*. *stercoralis* (SS)	0.839	[0.28 − 2.20]	0.12	0.73	
**TT x HW**	**12.394**	**[1.57 − 267.58]**	**5.91**	**0.015**	*
AL x TT	−	−	−	−	
AL x HW	−	−	−	−	
AL x TT x HW	−	−	−	−	

* p<0.05

** p<0.01

## Discussion

We found that infection by *Ascaris lumbricoides* (AL) and *Trichuris trichiura* (TT) were both independently associated with significant reductions in cerebral malaria risk, with TT conferring an estimated 90% protection from severe malaria. However, in addition to these direct effects we found that the interaction between two species, TT and hookworm (HW), also had consequences for the development of cerebral malaria. Specifically, the presence of HW nearly halved the protective effect of TT, revealing an intriguing indirect effect of HW on cerebral malaria potentially manifest through its interaction with TT. While the protective effects of single helminth species on cerebral malaria have been previously described [23,24,30,33], the indirect effect we observed in this study provides new evidence that the outcome of pairwise interactions between parasite species can depend on the presence of other parasites in the system. This new insight highlights how complex interactions occurring between parasite species can have non-intuitive consequences for infectious disease outcomes.

Our observation of a direct protective effect of *Ascaris lumbricoides* on cerebral malaria is in agreement with previous reports for this helminth species [29,30]. However, the association between cerebral malaria and *T*. *trichiura* has not been previously reported. The protection conferred by some helminths on severe malaria disease has been widely attributed to helminth-mediated reductions in immunopathology [30,34,35]. Although, the specific immunoregulatory mechanisms by which species like *A*. *lumbricoides* and *T*. *trichiura* confer protection from cerebral malaria are still not fully understood, the effect likely relates to a shift towards regulatory (Treg) and Th2-type lymphocytes which dampen the immune hyper-activation that underlies cerebral malaria [35,36]. For example, experimental infection with the helminth *Schistosoma mansoni* in baboons was shown to protect against severe malaria outcomes following inoculation with *Plasmodium knowlesi*, and immunological assays revealed that helminth-infected individuals showed fewer signs of overactive inflammatory responses [37].

Intriguingly, the protective effect of *Trichuris trichiura* on cerebral malaria was nearly halved in the presence of hookworm. Unlike many other helminth species, the human hookworm, *Necator americanus*, does not stimulate Th2-type immunity, rather it down-regulates the Th2 response [38]. Since *N*. *americanus* was likely the most common hookworm in our dataset, it is possible that concomitant hookworm infection dampened the immune-regulatory response triggered by *T*. *trichiura* thereby reversing some of the protection conferred by this species. Although the mechanistic details by which *Trichuris* and hookworm interact to affect cerebral malaria are largely speculative at this point, our result indicates that the co-occurrence of multiple helminth species can have cryptic and profound implications for disease severity.

The direct and indirect associations we found between helminth infections and malaria severity may have been influenced by a number of other risk factors that our data could not address. For example, variance among patients in malaria exposure history, socio-economic status, education level, or environmental factors that influence risk for both helminth infection and development of cerebral malaria could all conceivably change the interpretation of our results. Further research will be needed to understand how such factors, particularly immunological heterogeneity due to previous malaria exposure, might affect the patterns we describe here. Additionally, further studies in populations from different geographic regions could help elucidate how generalizable these associations are across diverse human and pathogen populations.

Co-infection with multiple helminth species is the norm rather than the exception in most hosts [39]. In our dataset, we identified a strong 3-way association between *A*. *lumbricoides*, *T*. *trichiura*, and hookworm, in which co-infection with all three species occurred more frequently than expected by random chance. This observation is in line with results from many other human populations [40–43]. Such positive associations between co-infecting helminths could reflect facilitative interactions between species, or may simply be a by-product of co-transmission given the similarity in the life cycle and transmission mode of soil-transmitted helminths in particular [44,45]. Irrespective of the mechanism underlying positive associations between helminth species, our work suggests that the co-occurrence of certain helminth species, in this case, *Trichuris trichura* and hookworm, can strongly shape the outcome of malaria infection. Given the commonness of multi-species helminth infections, these types of interactive effects of co-infecting helminths on microparasite infections may be more important than is currently recognized.

Indirect, or “context-dependent”, effects of environment, resources, or host traits have been repeatedly shown to impact parasite interactions [46,47], including the outcome of co-infection (e.g., [48–50]). Our analysis showing a cryptic indirect effect of one parasite on the interaction between two others suggests that the parasite community is another key “context” that can influence the outcome of within-host parasite interactions. In an empirical example of this, [51] showed that competitive outcomes among co-colonizing *Escherichia coli* strains in sterile culture were significantly altered when the experiments were conducted in the presence of natural human gut microbes. Taken together, these studies highlight the importance of moving from a pairwise to a multi-species perspective for understanding co-infection outcomes. Despite growing acceptance of the need for this host-as-habitat approach [52], fueled by the recent uptick in collection of high-throughput microbiome datasets in relation to a suite of transmissible and non-transmissible diseases, there is no consensus on tractable methods for doing so. Multivariate analyses (in particular, factor analysis and structural equation modeling) can help describe communities of organisms and their relationship with a focal infection. However, methods for identifying precise associations or testing interactions involving multiple species (e.g., those outlined in both [31] and [53,54]) continue to suffer from logistical, statistical, and computational limitations. Our study illuminates a compelling example of non-intuitive multi-species associations, adding to the mounting evidence that such methods are in great need of adoption for the study of parasite interactions.

The hypothetical framework used to explain interactions between gastrointestinal helminths and malaria has shifted over time from a nutritional one [55], to one based on immunosuppression/immunomodulation, in which all worms are pooled together for analysis [29], and finally to one where a distinction between immune mechanisms and hematological ones is made and analyses distinguish between worms with strong immunomodulatory effects (e.g. *Ascaris*) and those with strong effects on blood loss (e.g. hookworm) [56]. However, while hookworms have been regularly associated with increased malaria incidence [56], the present analysis identifies an even more complex negative effect of this worm on malaria outcomes. Thus, even though the development of a hookworm-specific vaccine could have benefits in terms of reducing overall malaria incidence, broad non-specific deworming programs that are not 100% efficient could have unexpected negative consequences in terms of malaria severity. Disease control programs must therefore consider the parasite community as a whole when evaluating the relative costs and benefits of specific control strategies, particularly in the tropics where high pathogen diversity and co-infections are common.

### Data, code and materials

The datasets supporting this article have been uploaded as part of the supplementary material.

## Supporting information

S1 Table Helminth species infection status and cerebral malaria occurrence counts for all hyperparasitemic P. falciparum cases. (PDF)

S1 Appendix Quantifying interspecific associations. (PDF)

S2 Appendix Associations among helminth species in young adults. (PDF)

S3 Appendix Multiple correspondence analysis. (PDF)
