# Supplementary material for: Disentangling complex parasite interactions: Protection against cerebral malaria by one helminth species is jeopardized by co-infection with another
Source: PLoS Negl Trop Dis. 2018 May 10;12(5):e0006483. doi: 10.1371/journal.pntd.0006483 (PMC5963812; doi:10.1371/journal.pntd.0006483)
Supplement: S1 Table — (PDF) [file pntd.0006483.s001.pdf]

Disentangling complex parasite interactions: protection against cerebral malaria by one helminth species is jeopardized by co-infection with another

Jessica L. Abbate, Vanessa O. Ezenwa, Jean-François Guégan, Marc Choisy, Mathieu Nacher, Benjamin Roche

Table S1: Helminth species infection status and cerebral malaria occurrence counts for all hyperparasitemic *P. falciparum* cases

Infection and Co-infection Frequencies of Hyperparasitemic *P. falciparum* patients with and without cerebral malaria (CM)							
Coinfection Status	Pathogen species present				Number of Occurrences	With CM	Without CM
4 species	*Al*	*Tt*	HW	*Ss*	4	1	3
3 species	*Al*	*Tt*	HW		17	1	16
	Al	*Tt*		*Ss*	1	0	1
	Al		HW	*Ss*	2	0	2
		Tt	HW	*Ss*	3	1	2
2 species	*Al*	*Tt*			11	0	11
	Al		HW		9	0	9
	Al			*Ss*	1	0	1
		Tt	HW		14	3	11
		Tt		*Ss*	1	0	1
			HW	*Ss*	6	1	5
Single infections	*Al*				9	1	8
		Tt			19	1	18
			HW		30	8	22
				Ss	12	3	9
Not infected					144	47	97
Sum					283	67	216

Table S1. Helminth species infection status and cerebral malaria occurrence counts for all hyperparasitemic *P. falciparum* cases. Helminth species included *Ascaris lumbercoides* (*Al*), *Trichuris trichuira* (*Tt*), hookworm (HW), and *Strongyloides stercoralis* (*Ss*).
