# Supplementary material for: Disentangling complex parasite interactions: Protection against cerebral malaria by one helminth species is jeopardized by co-infection with another
Source: PLoS Negl Trop Dis. 2018 May 10;12(5):e0006483. doi: 10.1371/journal.pntd.0006483 (PMC5963812; doi:10.1371/journal.pntd.0006483)
Supplement: S1 Appendix — (PDF) [file pntd.0006483.s002.pdf]

Disentangling complex parasite interactions: protection against cerebral malaria by one helminth species is jeopardized by co-infection with another

Jessica L. Abbate, Vanessa O. Ezenwa, Jean-François Guégan, Marc Choisy, Mathieu Nacher, Benjamin Roche

Appendix S1: Quantifying interspecific associations

Methods

The basic statistic evaluated for this method of quantifying interspecific association is significance according to the association screening (SCN) analysis, described in detail by Vaumourin *et al*. (2014). This analysis uses individual-based presence-absence information for *n* species infecting a single group of hosts to identify associations among species from infection surveillance data. The assumptions made are that the host population is homogeneous, that the sampling is not biased towards presence or absence of each parasite species, and that the number of hosts tested must exceed the number of presence-absence combinations possible (NC = 2^n) to achieve adequate power. Rarefaction curves of this statistic, described in detail below, were then generated for each combination of parasite presence/absence possible. These rarefaction curves can then be compared to determine the relative strength of associations across the range of possibilities.

Rarefaction Procedures

1. Define the number of data partitions (NDP) to be sub-set. Here, we partitioned the dataset into 10 subsets containing 10% to 100% of the original data, increasing by increments of 10 (resulting in 10 ordered partitions, NDP=10).

2. Define the number of SCN analysis repetitions to be conducted on each partition. Here, we used 20 repetitions for each of the 10 partitions.

3. For each partition, generate the appropriate number of subsets by randomly sampling from to full dataset always with replacement. Here, this was 20 random non-exclusive subsets of the data for each of the 10 partitions.

4. For each random subset (20 per partition x 10 partitions = 200 subsets), run the screening analysis.
   The SCN analysis itself runs 5000 simulations using the prevalence and number of individual hosts (specific to each subset) to come up with a 95% confidence interval for the null hypothesis (random associations, based on Bernoulli probability distribution), and gives each association combination a score for being outside of the 95% confidence envelope (1 or 0). A p-value is then also calculated based on the number of simulations where the expected (given random associations) number of individuals with each combination (*x*) is beyond (above or below) the

observed value (*xobs*) (p = 2 x number of simulations where ($x > xobs$) /number of simulations). This p-value is based on a confidence interval derived from discrete counts, so it should be used with caution, but it does not need to be corrected for inflation of type I error due to multiple hypothesis testing. Typically, any associations consistently found beyond the confidence envelope of expected frequency have a p-value of 0 or less than 10^{-4}. The p-value for each combination in each subset is recorded. Because the SCN analysis is based on random simulations of the null expectations, even results conducted repeatedly using 100% of the data could potentially differ.

5. To decide whether each combination is "positive for an association" under each partition (e.g., if co-infection combination “10110” in the 20% data partition = ✓), a user-defined rule determines if support for the association is relatively stronger or weaker. The rule requires the user to set the significance level (SL) and the frequency of detection (FD), both defined below. For the current analysis, the rule was as follows (SL=0.05, FD=95%):
   Each combination under each partition is positive for an association IFF it has a p-value of less than SL (0.05) in FD (95%) of random subsets (here, at least 19 of 20 subsets). The p-value (SL) indicates the desired strength of the significance level, and the frequency with which that significance is achieved (FD) among the number of simulations (NS) indicates how robust the association is to error from sub-setting the data for that partition. Associations with the strongest support would be detected only when SL=0 and FD=100. Weaker but robust associations might be detected when SL=0.05, FD=100. The reason for manipulating the strength of the association detected is that when the rules are too stringent, there is much less subtlety in the results, making it difficult to detect differences between e.g., the strength of combinations 10000 vs. 01000 vs. 00100 to infer which parasite might be responsible for driving the positive 3-way association (11100).

6. For each combination in each partition, the maximum p-value is recorded. The maximum p-value considering 100% of the data is reported as “SCN p-value <=”, indicating the most conservative estimate for the probability that the association detected in the dataset occurred by random chance alone. Using the last (20th) run of the SCN analysis from partition considering 100% of the data, the “direction” of the association (more rare or frequent than expected by random chance) is reported. If the observed frequency of occurrence is below the midpoint of the confidence interval, its direction is recorded as “rare”; if above the midpoint, it is recorded as “frequent”; if it is within 0.5 occurrences of the midpoint, it is recorded as “random”.

7. Finally, a “rarefaction robustness” (RR) score for the association detected for each co-infection combination is given. The score ranges from 0 to 10, and indicates the number of data partitions in which a significant association is detected and robust, given the respective significance level

(SL, 0.05) and frequency of detection (FD, 95%) specified. The larger the robustness score, the stronger the association given equal maximum p-values. RR=1 indicates that 100% of the data are needed (robust association not detected when the dataset is reduced) while RR=10 indicates that the association was detected in all 10 partitions (only 10% of the data are needed); RR=0 indicates that the combination did not fulfill the criteria for a robust association.

References

Vaumourin, E., Vourc'h, G., Telfer, S., Lambin, X., Salih, D., Seitzer, U., *et al.* (2014). To be or not to be associated: power study of four statistical modeling approaches to identify parasite associations in cross-sectional studies. *Front. Cell. Infect. Microbiol.*, 4, 1–11
