# Supplementary material for: Disentangling complex parasite interactions: Protection against cerebral malaria by one helminth species is jeopardized by co-infection with another
Source: PLoS Negl Trop Dis. 2018 May 10;12(5):e0006483. doi: 10.1371/journal.pntd.0006483 (PMC5963812; doi:10.1371/journal.pntd.0006483)
Supplement: S2 Appendix — (PDF) [file pntd.0006483.s003.pdf]

Disentangling complex parasite interactions: protection against cerebral malaria by one helminth species is jeopardized by co-infection with another

Jessica L. Abbate, Vanessa O. Ezenwa, Jean-François Guégan, Marc Choisy, Mathieu Nacher, Benjamin Roche

Appendix S2 : Associations among helminth species in young adults

Results

The MCA analysis (Appendix S3 Figure A3.3(b)) showed that infection by AS, TT, and HW differed among age groups. We explored this caveat and found that age distributions of patients infected by the three species were similar in shape but not identical (Figure A3.1). Nonetheless, SCN and rarefaction analysis performed after restricting the dataset to the most infected age group (15-20 years old, N=90) yielded a similar pattern of helminth species associations despite the reduced power (Table S3.1).

Figure A2.1. Age distributions of (a) all hyperparasitemic malaria patients in the study, and of those infected with either (b) *Ascaris lumbercoides*, (c) *Trichuris trichiura*, or (d) hookworm (*Necator americanus*). Age is given in years.

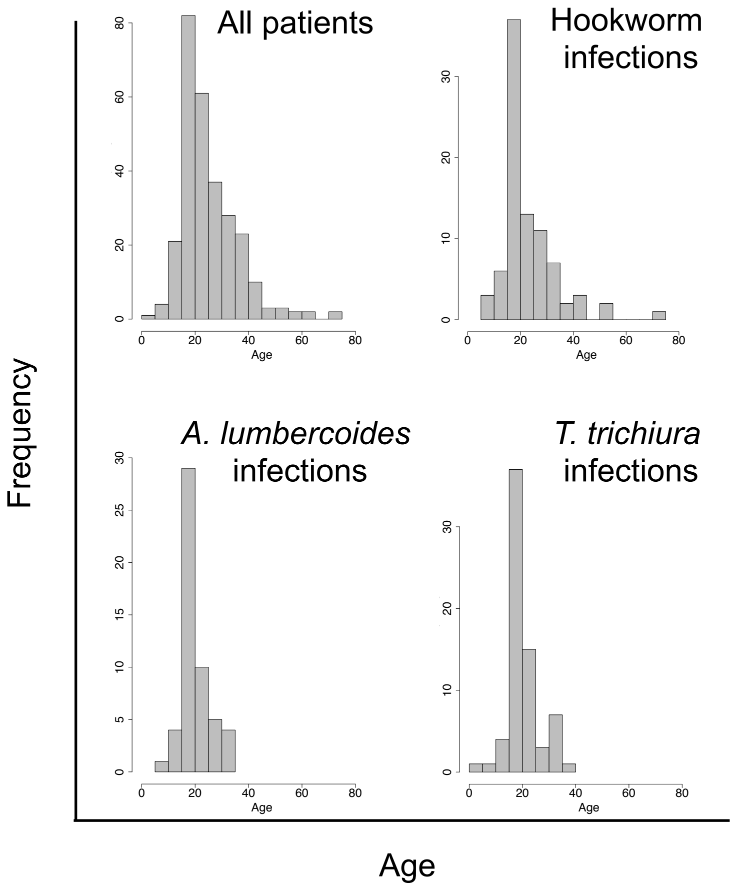

Table A2.1. Association screening (SCN) analysis results for all detected helminth species infecting hyperparasitemic malaria patients between the ages of 15 and 20, inclusive (N=90). SCN P-values reported are the maximum p-value returned by the SCN analysis when 100% of the data are sampled. Significant or trending associations detected are in bold for emphasis.

SCN and Rarefaction Results					All hyperparasitemic malaria patients between 15-20 years old (N=90)			
Coinfection Status	Pathogen species present				Direction	Percentage of data sampled 10% ➔ 100%	Robustness Score	SCN P-value <=
4 species	***Al***	***Tt***	**HW**	***Ss***	**Random**		**0**	**0.0880**
3 species	***Al***	***Tt***	**HW**		**Frequent**	✓	**1**	**0.0204**
	Al	*Tt*		*Ss*	Rare		0	1.0000
	Al		HW	*Ss*	Random		0	0.1512
		Tt	HW	*Ss*	Rare		0	0.9484
2 species	*Al*	*Tt*			Rare		0	0.6944
	Al		HW		Rare		0	0.5436
	Al			*Ss*	Rare		0	1.0000
		Tt	HW		Rare		0	0.9984
		Tt		*Ss*	Rare		0	0.9392
			HW	*Ss*	Rare		0	0.8780
Single infections	***Al***				**Rare**		**0**	**0.0988**
		Tt			Rare		0	0.1552
			**HW**		**Rare**		**0**	**0.0796**
				Ss	Rare		0	0.7000
Not infected					**Frequent**	✓	**1**	**0.0012**
