# Supplementary material for: Disentangling complex parasite interactions: Protection against cerebral malaria by one helminth species is jeopardized by co-infection with another
Source: PLoS Negl Trop Dis. 2018 May 10;12(5):e0006483. doi: 10.1371/journal.pntd.0006483 (PMC5963812; doi:10.1371/journal.pntd.0006483)

Disentangling complex parasite interactions: protection against cerebral malaria by one helminth species is jeopardized by co-infection with another

Jessica L. Abbate, Vanessa O. Ezenwa, Jean-François Guégan, Marc Choisy, Mathieu Nacher, Benjamin Roche

Appendix S3: Multiple correspondence analysis

Methods

Multiple correspondence analysis (MCA) was used to understand how the gastrointestoinal helminth community of hyperparasitemic *P. falciparum* patients was structured in order to reduce the number of variables accounting for differences among individuals. In addition to severe malaria outcome, composition of the helminth community was examined for sex and age groups. The analysis was implemented in the R statistical computing environment (R Core Team & R Development Core Team 2011) using the 'MCA' function in package *FactoMineR* (Lê *et al.* 2008) and visualized using tools available from the *factoextra* package. The raw data input was a presence/absence table with a 1 or 0 marking infection by each of the four helminth species *Ascaris lumbercoides* (*Al*), *Trichuris trichuira* (*Tt*), *Necator americanus* (hookworm, HW), and *Strongyloides stercoralis* (*Ss*); in columns) for each patient (in rows). Logistic regression was carried out to assess the contribution of each resulting MCA dimension after first correcting for patient sex, age group, and all other MCA dimensions using the 'glm' function with a binomial logit link from the *stats* package, and the 'drop1' function (setting the unordered contrasts option to "contr.sum") for χ^2 analysis of deviance.

Results

The MCA resulted in 4 orthogonal dimensions, each explaining diminishing proportion of the variance in infection/co-infection frequencies among individuals (Dim1: 42.8%; Dim2: 24.9%; Dim3: 17.5%, Dim4: 14.8%; Figure A3.1(a)). The first two dimensions explained a cumulative 67.7% of variation in infection/co-infection occurrences. Helminth communities varied between patients that did or did not develop cerebral malaria (Figure A3.2(a)), and among age classes (Figure A3.3(b)), but were nearly identical between sexes (Figure A3.3(a)). After controlling for sex and age class, Dim1 (defined overwhelmingly by the presence or absence of *Al*, *Tt*, and HW; Figure A3.4(c)) was highly significant for defining differences in the helminth community infecting patients that did versus those that did not develop cerebral malaria (p=0.00017; Table A3.1; Figure A3.4(a)). Dimension 3, defined primarily by the presence of HW, was the only other variable that showed any trend for an effect of helminth species on developing cerebral malaria (at p=0.11; Table A3.1; Figure A3.4(b)).

Conclusion

These results revealed that *Al*, *Tt*, and HW – and potential interactions among them – are the most important contributors to helminth-related protection from

cerebral malaria. Overlapping but incongruent influence of helminth species on Dim1 and Dim3 (Figure A3.1(c)) point to likely interaction effects.

Table A3.1. Logistic regression analysis of helminth community, defined by multiple correspondence analysis (MCA) dimensions (Dim1-Dim4) on the risk of developing cerebral malaria. Statistical significance for each explanatory variable was determined by log-likelihood ratio tests after correcting for all other factors in the model using $\chi 2$ analysis of deviance.

	Adjusted odds of developing cerebral malaria outcome			
	Estimate	95% CI	Deviance $\chi 2$	p-value
Intercept	0.249	[0.16 – 0.38]		
Sex			0.50	0.48
F(75) vs. M(204)	0.883	[0.61 – 1.24]		
Age class			0.35	0.84
<20(88) vs. 20-40(166)	0.864	[0.51 – 1.44]		
<20(88) vs. 40+(25)	1.062	[0.70 – 1.63]		
MCA Dim1	0.329	[0.15 – 0.61]	**14.17**	**0.00017**
MCA Dim2	1.398	[0.71 – 2.66]	0.99	0.32
MCA Dim3	0.537	[0.24 – 1.15]	2.56	0.11
MCA Dim4	0.719	[0.26 – 1.86]	0.45	0.50

Figure A3.1 Multiple correspondence analysis (MCA) results for helminth community composition are described by (a) the contribution of each dimension to the overall variance in the data, (b) variable correlations with the first two dimensions of the MCA, and (c) variable contributions to each orthogonal MCA dimension.

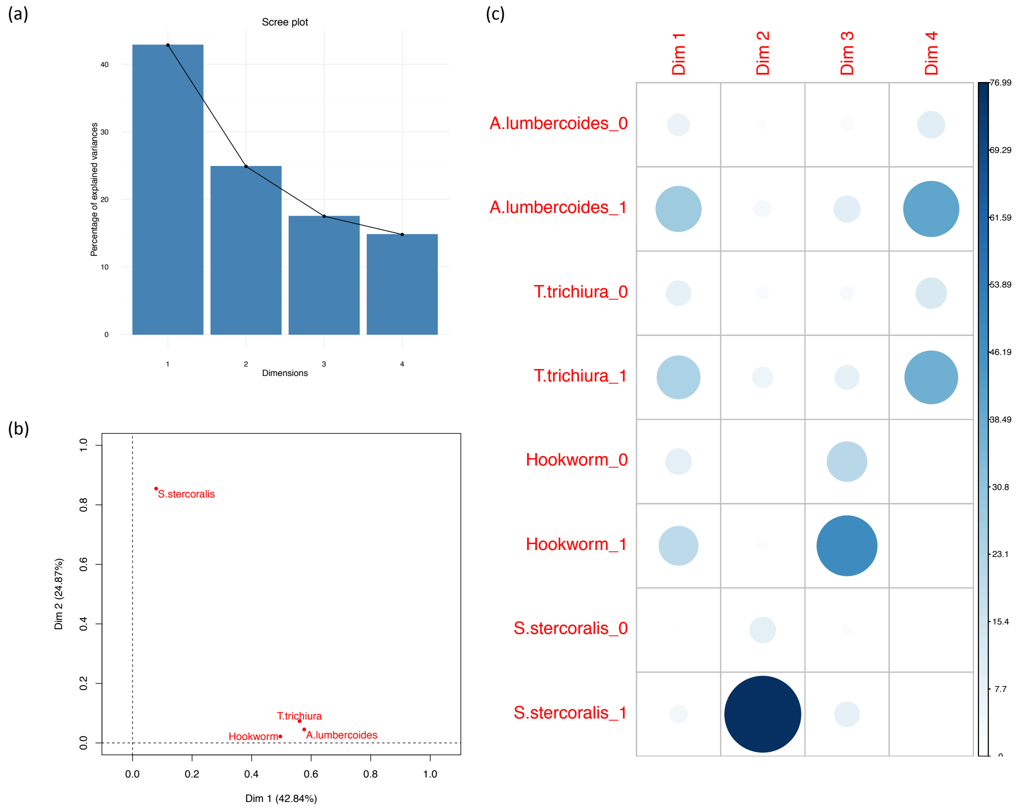

Figure A3.2 Helminth community composition among hyperparasitemic *P. falciparum* infections that did (red) and did not (blue) lead to severe (cerebral) malaria outcome, represented by plotting the mean values for the (a) first and second (accounting for 56% of total variance in the data) or (b) first and fourth dimensions (accounting for the two most important variables) determined by multiple correspondence analysis (MCA). Ellipses include 95% of individual values for each class of patients.

Helminth community composition between cerebral and non-cerebral malaria cases

(a) MCA Dim1 vs. MCA Dim2 (b) MCA Dim1 vs. MCA Dim3

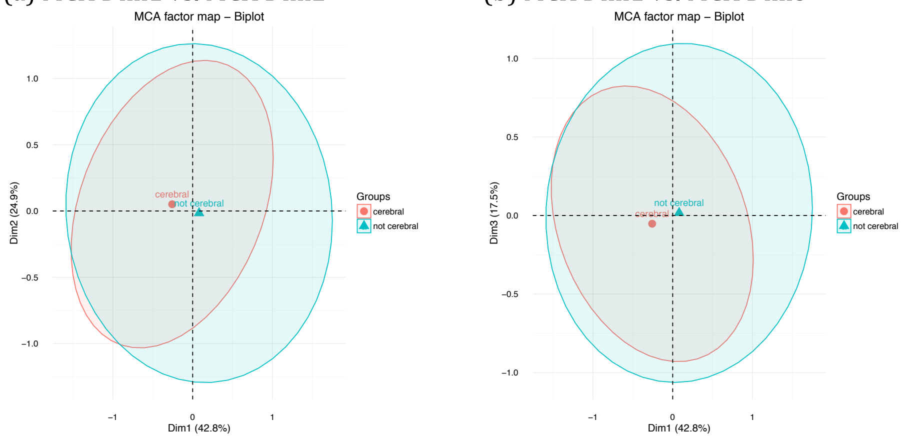

Figure A3.3 Helminth community composition among (a) females and males or (b) age classes with hyperparasitemic *P. falciparum* infection, represented by plotting the mean values of the first and second dimensions determined by multiple correspondence analysis (MCA) (accounting for 68% of total variance in the data). Ellipses include 95% of individual values for each class of patients.

Helminth community composition among sexes and age classes (MCA Dim1 vs. MCA Dim2)

(a) sexes (b) age groups

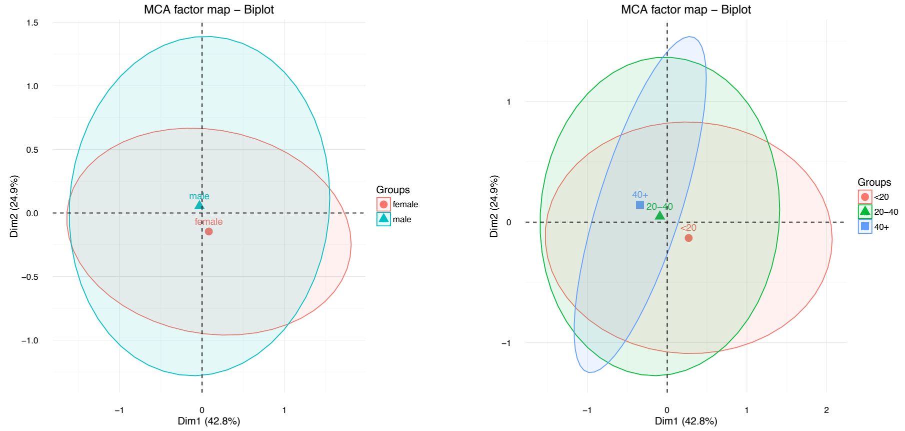

Figure A3.4 Helminth community differences among patients with hyper-parasitemic *P. falciparum* infection that did or did not develop cerebral malaria, represented by values of the (a) first and (b) fourth dimensions determined by multiple correspondence analysis (MCA). Dim1 was influenced most heavily by the presence of *Al*, *Tt*, and HW; Dim3 was influenced most heavily by the presence of HW. Univariate t-test results are given above each plot, and asterisks (***) indicate significance of p<0.001.

(a) MCA Dim1

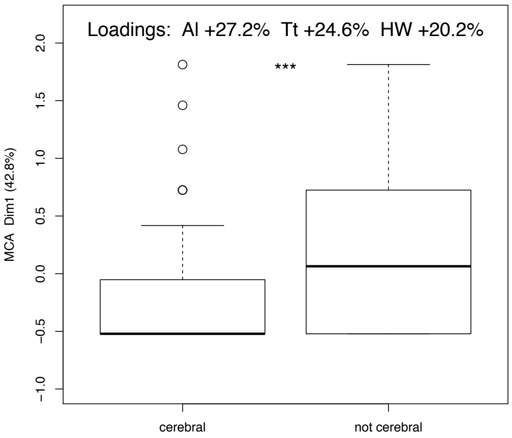

(b) MCA Dim3

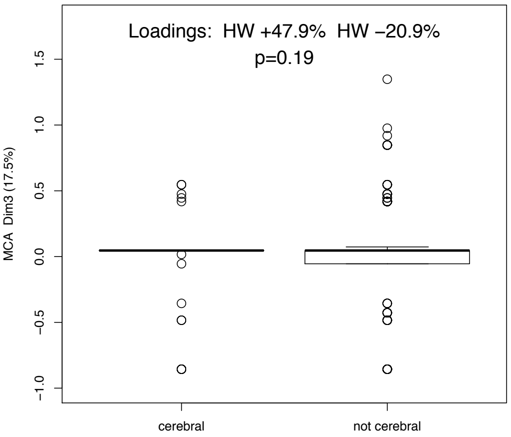

Supplement: S3 Appendix — (PDF) [file pntd.0006483.s004.pdf]
